# Cluster of Human Tanapox Cases in Wildlife Reserve, South Africa, 2024

**DOI:** 10.3201/eid3207.251961

**Published:** 2026-07

**Authors:** Monica Birkhead, Antoinette A. Grobbelaar, Daniel Morobadi, Hlupi D. Mpangani, Julia Dabrowski, Kamini Govender, Lucille H. Blumberg, Mandla Zwane, Naazneen Moolla, Naume D. Tebeila, Nevashan Govender, Terry Marshall, Veerle Dermaux-Msimang, Wayne Grayson, Jacqueline Weyer

**Affiliations:** National Institute for Communicable Diseases, Johannesburg, South Africa (M. Birkhead, A.A. Grobbelaar, K. Govender, L.H. Blumberg, N. Moolla, N.D. Tebeila, N. Govender, V. Dermaux-Msimang, J. Weyer); Ampath Laboratories Ltd., Centurion, South Africa (D. Morobadi, T. Marshall, W. Grayson); Mpumalanga Department of Health, Mbombela, South Africa (H.D. Mpangani, M. Zwane); Hoedspruit Family Practice, Hoedspruit, South Africa (J. Dabrowski); Right to Care, Johannesburg (L.H. Blumberg); University of Stellenbosch, Stellenbosch, South Africa (L.H. Blumberg); University of Pretoria, Pretoria, South Africa (L.H. Blumberg, N. Moolla, J. Weyer); University of the Witwatersrand, Johannesburg (N. Moolla, W. Grayson, J. Weyer)

**Keywords:** Tanapox virus, Poxviridae, viruses, zoonoses, vector-borne infections, cutaneous lesions, wildlife-human interface, South Africa

## Abstract

Tanapox is a rare, self-limiting, mosquitoborne viral zoonosis. During February–March 2024, we identified 11 human tanapox cases near Orpen in Kruger National Park, South Africa. We retrospectively identified 2 suspected cases from Pafuri from 2021, suggesting continued virus circulation. Public awareness of tanapox is essential for appropriate medical treatment.

Tanapox virus (TANV), a member of the genus *Yatapoxvirus* (Poxviridae), is associated with a rare zoonotic infection; only 1 human tanapox case has been reported globally since 2004 (*1**–**3*). That case originated in Skukuza, a subtropical area of Kruger National Park (KNP), a wildlife reserve in South Africa. That geographic location differed from all previously recorded tanapox cases from endemic countries in equatorial Africa, including Democratic Republic of the Congo, Kenya, Republic of Congo, and Sierra Leone, and from Tanzania in tropical east Africa (*4**–**9*). Skukuza is also south of the possible TANV distribution predicted by ecologic niche modeling (*9*).

Although geographic origins differ, the environmental and clinical features of all reported human tanapox cases are comparable. TANV is thought to be transmitted mechanically from wildlife hosts (nonhuman primates) to humans by hematophagous culicine mosquitoes (*4**–**10*). All previously reported cases originated from locations where human–wildlife interfaces were closely juxtaposed, after heavy rainfall and high temperatures that preceded the mosquito breeding season (*3**–**5*,*9*).

Generally, TANV infection presents as 1–3 nodules in exposed body areas where mosquitoes typically tend to bite, such as hands, elbows, lower limbs, and toes (*5*). Although the centrally umbilicated lesions are self-limiting, resolving without intervention over 6–8 weeks, TANV infection can cause symptoms consistent with a viraemia, including fever, fatigue, intense headaches, myalgia, lymphadenopathy, and viral exanthem. The lesions can be unsightly, painful, and pruritic and infrequently become secondarily infected after scratching (*2*,*4*,*5*,*8*). As yet, no human-to-human TANV transmission or infection from contact or fomites has been recorded, and no deaths or systemic infection have been reported (*2*,*5*,*11*). Given the self-limiting nature and mildness of the infection, underreporting by workers in wildlife areas is highly probable, but for persons who do seek medical assistance, a lack of familiarity with the disease could lead to misdiagnosis and inappropriate treatment. We describe a cluster of tanapox cases from in and around KNP, South Africa, a novel, subtropical location for TANV transmission.

## The Study

**Figure 1:**
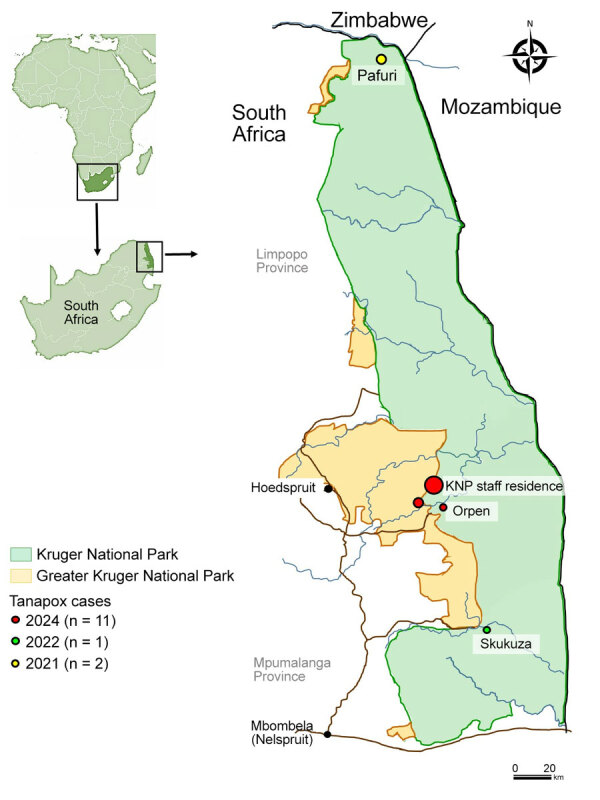
Locations of cases in a cluster of human tanapox cases in wildlife reserve, South Africa, 2024. Of 11 cases in 2024, nine were laboratory confirmed by PCR and sequencing, and 2 were suspected on the basis of clinical symptoms and epidemiologic links. In addition, one case was laboratory-confirmed in 2022. We also retrospectively identified 2 suspected cases from 2021 on the basis of lesion features, clinical symptoms, and suitable environmental parameters. Insets show location of KNP within South Africa and the continent. KNP, Kruger National Park.

In February 2024, a patient (case 1) with several skin lesions sought care from a general practitioner in Hoedspruit, a small town ≈68 km west of KNP. The patient resided in KNP staff lodging near Orpen (Figure 1). On the advice of an infectious diseases expert familiar with poxvirus infections, a lesion swab sample was sent to the Special Viral Pathogens Laboratory, Centre for Emerging Zoonotic and Parasitic Diseases, National Institute for Communicable Diseases (NICD), Johannesburg, South Africa, for poxvirus testing.

Using previously described methods (*3*), we performed molecular testing to identify the genus of poxvirus involved. We confirmed *Yatapoxvirus* TANV by sequencing, which we corroborated by transmission electron microscopy of a subsequent lesion biopsy (Table; Appendix Figure 1).

**Table:** Epidemiologic data of patients in a cluster of human tanapox cases in wildlife reserve, South Africa, 2024

Case no.	Age, y/sex	Location	Symptom onset date	No. lesions	Body part affected	Laboratory-confirmed*
1	12/M	KNP staff residence	Feb 8	8	Elbow, hand, finger	Y
2	33/F	KNP staff residence	Feb 8	1	Shin	Y
3	43/F	KNP staff residence	Feb 19	1	Knee	Y
4	41/M	KNP staff residence	Early Feb	2	Fingers	N
5	38/M	KNP staff residence	Feb 10	1	Back of knee	Y
6	34/M	KNP staff residence	Feb 19	1	Big toe	Y
7	58/M	KNP staff residence	Feb 27	1	Knee	Y
8†	47/M	Open camp, KNP	Mar 3	3	Elbow, hand	Y
9	33/M	KNP staff residence	Mar 13	1	Hand	Y
10‡	>30/M	Private camp, greater KNP	Unknown	Unknown	Unknown	N
11‡	>30/M	Private camp, greater KNP	Unknown	Unknown	Unknown	Y

*Confirmed by PCR and sequencing of dry swab sample of lesion, following previously described methods (*3*). KNP, Kruger National Park. †Lesion swab sample accompanied by skin punch biopsy for electron microscopy. ‡No complete questionnaire submitted with lesion swab sample.

Two other persons (cases 2 and 3), household members of case-patient 1, also had pox-like nodules (Table). Consequently, we developed and distributed a questionnaire through established, community communication networks that link KNP and Greater KNP, a collection of private and community-owned wildlife reserves adjacent to the western boundary of KNP. We received 10 additional lesion swab samples (cases 2–11) for molecular confirmatory testing, among which 8 (cases 2–9) were accompanied by a completed questionnaire (Table). Of those 10 lesion swab samples, 8 were TANV-positive, and we submitted a representative example of the sequences to GenBank (accession no. PV816105). Two specimens, from cases 4 and 10, failed PCR amplification because the swabs were likely collected after the lesions had sufficiently healed and consequently no longer contained viral DNA. However, we included those 2 as suspected cases on the basis of epidemiologic and clinical features common to all 11 cases in the cluster.

**Figure 2:**
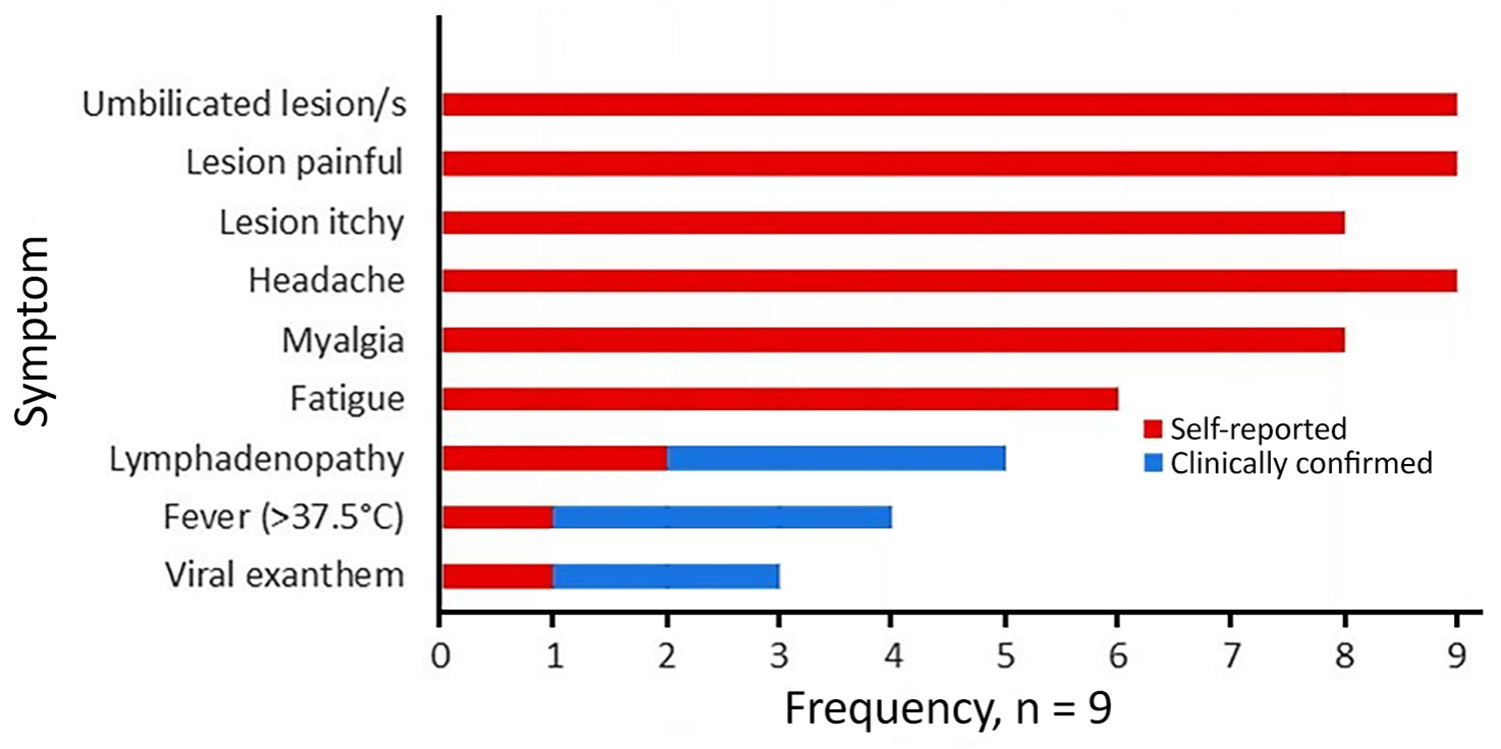
Clinical data from a cluster of human tanapox cases in wildlife reserve, South Africa, 2024. Data represent 9 laboratory-confirmed cases of 11 persons for whom lesion samples were submitted.

All TANV cases occurred within 20 km of each other (Figure 1). Most case-patients were male (7 game rangers, 1 adult visitor, and 1 resident child); 2 were female (both KNP residents). Lesions and symptoms were consistent with those described in cases originating from equatorial Africa during 1957–2004 (*4**–**8*). Typically, case-patients had 1–3 umbilicated lesions coinciding with common sites for mosquito bites (Table). Case-patients noted that nodules initially were itchy; some mentioned that they thought nodules were just mosquito or insect bites, but most case-patients said nodules later became painful (Figure 2). Case-patients reported other symptoms of TANV infection, most consistently headaches, myalgia, and fatigue (Figure 2). Among 3 case-patients who reported a maculopapular viral exanthem, 2 described a generalized distribution over the torso.

An initial misdiagnosis of impetigo in some patients prompted topical antibiotic treatment, and 1 person was treated with intravenous antibiotic drugs for a suspected superinfection associated with lymphadenitis. Lesion development described in questionnaires by 2 persons detailed the progression from an itchy bite to a firm, circular nodule with an erythematous base (Appendix Figure 2). The centrally umbilicated nodules, some of which enlarged to 20 mm diameter, remained firm and noncystic. Other nodules ulcerated at a diameter of <15 mm, forming craters with raised edges around a necrotic central area. Subsequent crusting and re-epithelialization began around the circumference, and concurrent tissue granulation developed in the center. About 25 days after symptom onset, the central tissue dried to form a scab, which darkened and sloughed off ≈3 weeks later. The initial erythematous areola surrounding each lesion persisted for the duration of lesion development, with varying degrees of associated edema and inflammation. Extensive inflammation, for example affecting an entire forearm (Appendix Figure 2), was more likely to be associated with local lymphadenopathy.

NICD compiled tanapox information for online public dissemination (*12*). NICD also alerted regional stakeholders, including the provincial health department, private practitioners, managers of wildlife reserves, and training facilities in the area, and asked them to further distribute tanapox information.

Serendipitous feedback included the possibility of 2 tanapox cases acquired by visitors to the Pafuri area of KNP in February 2021 (Figure 1). On telephonic follow-up, we suspected clinical diagnoses of tanapox in the 2 adult male visitors on the basis of a suitable environment (omnipresent nonhuman primates and a perceived abundance of mosquito vectors after high rainfall recorded in previous weeks), lesion attributes (photographic evidence of number, placement, and appearance), as well as other recalled symptoms, including headaches, myalgia, and extreme fatigue. Both men sought medical assistance; 1 had his lesion excised, and the other was placed on intravenous antibiotic drugs because of apparent purulence of 1 of the lesions, possibly associated with a diabetically compromised immune system. 

## Conclusions

This cluster of cases strongly suggests autochthonous TANV transmission is occurring in the KNP area. Therefore, residents, workers, and visitors to KNP and surrounding areas need to be aware of the possibility of tanapox during February and March, after peak summer rainfall. Because no evidence supports the assumption that inflammation associated with tanapox nodules is caused by a bacterial superinfection, clinicians should be aware of tanapox symptoms and pathogenesis and avoid antibiotic use. Instead, clinicians can provide more applicable patient management by reducing anxiety, unnecessary medical procedures, and expense. As with any mosquito-transmitted virus, persons at risk for mosquito exposure should follow recommendations for using mosquito repellents and bed nets, especially considering that no antiviral drugs or vaccines for TANV are available. 

Appendix Additional information cluster of human tanapox cases in wildlife reserve, South Africa, 2024.
