# Supplementary material for: Cluster of Human Tanapox Cases in Wildlife Reserve, South Africa, 2024
Source: Emerg Infect Dis. 2026 Jul;32(7):1159–62. doi: 10.3201/eid3207.251961 (PMC13322442; doi:10.3201/eid3207.251961)
Supplement: Appendix — Additional information cluster of human tanapox cases in wildlife reserve, South Africa, 2024. [file 25-1961-Techapp-s1.pdf]

Article DOI: https://doi.org/10.3201/eid3207.251961

EID cannot ensure accessibility for supplementary materials supplied by authors. Readers who have difficulty accessing supplementary content should contact the authors for assistance.

Cluster of Human Tanapox Cases in Wildlife Reserve, South Africa, 2024

Appendix

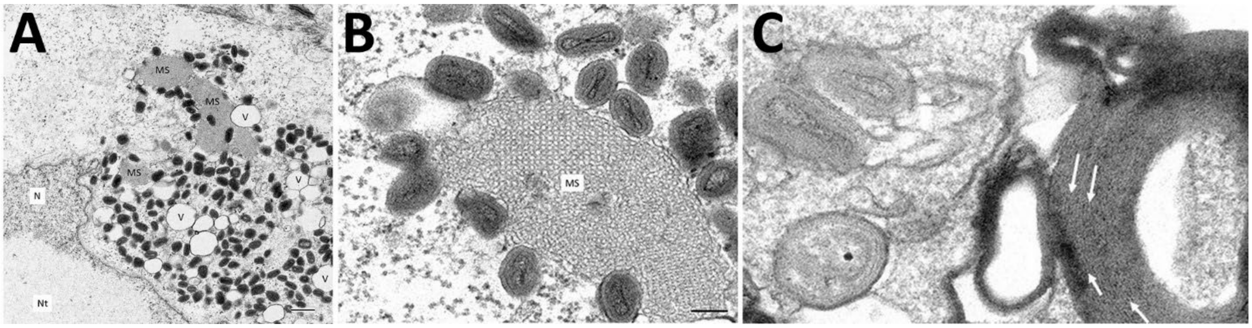

Appendix Figure 1. Electron micrographs illustrating TANV ultrastructural features that enable the differentiation of TANV from species within the *Orthopoxvirus* genus. A) Nuclei (N) typically have a central electron-lucent region (Nt). Mature virions are not found in finely granular inclusion bodies, and are associated with extensive membranous structures (MS) and bubble-like clusters of vacuoles (V). Scale bar indicates 500 nm. B) Membranous structure surrounded by virions. Scale bar indicates 150 nm. C) Infected keratinocytes have many osmiophilic inclusions associated with finely lamellated structures (arrows). Scale bar indicates 120 nm.

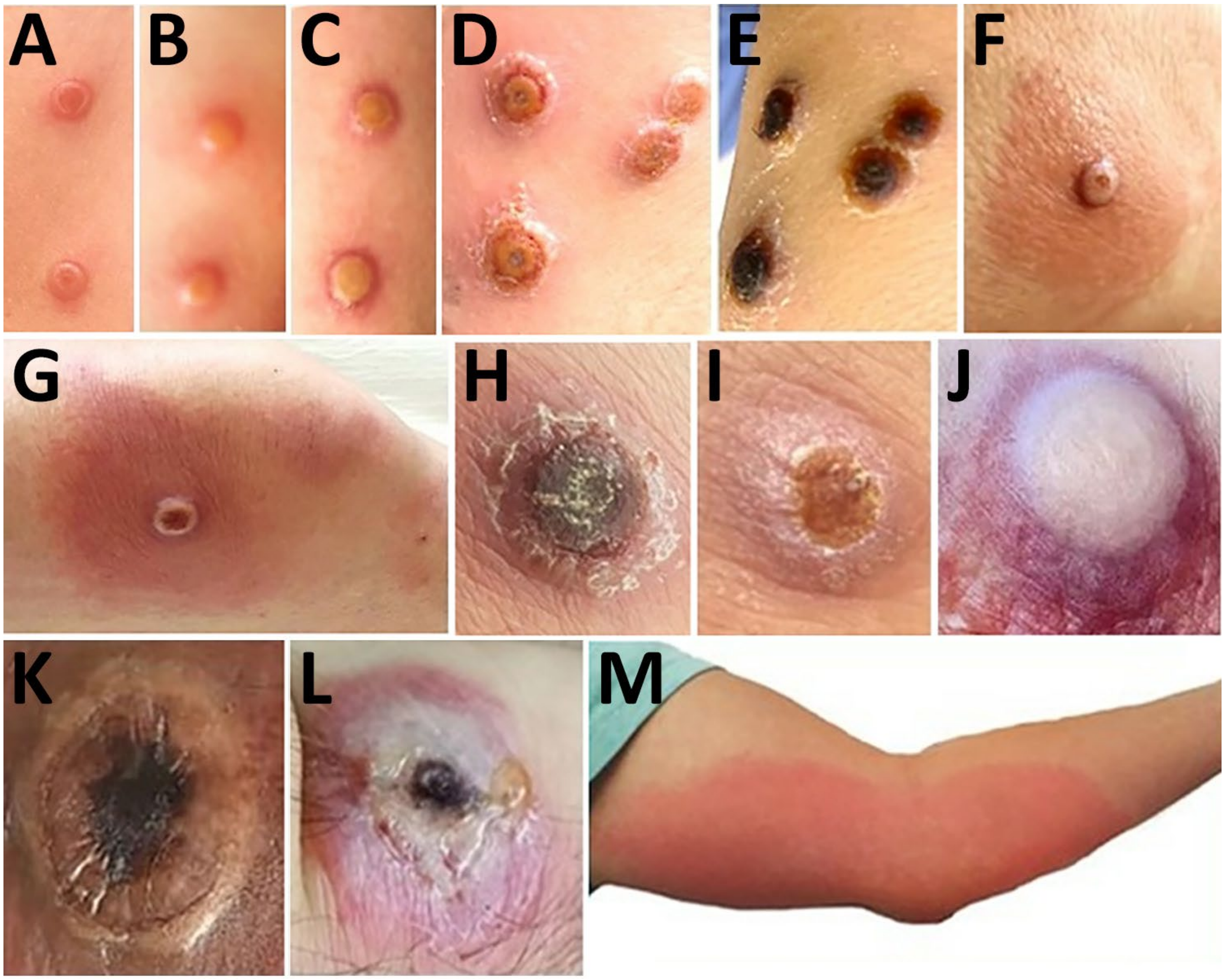

Appendix Figure 2. Tanapox lesions from case-patients, South Africa. A–E) Progressive lesion development (nodular, ulcerative, crusting) on the forearm, on days 1, 3, 5, 10, and 14. Note the erythematous areola around each lesion. F–I) Lesion development on the knee on days 1, 7, 13, and 21, with associated inflammation. J) Nodular lesion with initiation of central umbilication. K) Classic tanapox lesion with central necrosis and crusting. L) Serous exudate from a lesion in the crook of the elbow, associated with extensive inflammation (M) and lymphadenopathy.
